# Sphingolipid Effects on the Plasma Membrane Produced by Addition of Fumonisin B1 to Maize Embryos

**DOI:** 10.3390/plants9020150

**Published:** 2020-01-23

**Authors:** Nora A. Gutiérrez-Nájera, Mariana Saucedo-García, Liliana Noyola-Martínez, Christian Vázquez-Vázquez, Silvia Palacios-Bahena, Laura Carmona-Salazar, Javier Plasencia, Mohammed El-Hafidi, Marina Gavilanes-Ruiz

**Affiliations:** 1 Instituto Nacional de Medicina Genómica. Periférico Sur 4124, Torre 2, 5° piso. Álvaro Obregón 01900, Cd. de México, Mexico; ngutierrez@inmegen.gob.mx; 2 Instituto de Ciencias Agropecuarias, Universidad Autónoma del Estado de Hidalgo, Avenida Universidad Km. 1, Rancho Universitario, Tulancingo-Santiago, Tulantepec, Tulancingo 43600, Hidalgo, Mexico; mariana_saucedo@yahoo.com.mx; 3 Departamento de Bioquímica, Facultad de Química, UNAM. Cd. Universitaria. Coyoacán 04510, Cd. de México, Mexico; lilianasofia27@gmail.com (L.N.-M.); christianavazquezv@comunidad.unam.mx (C.V.-V.); silvia_palacios@itmilpaalta.edu.mx (S.P.-B.); carmonal@comunidad.unam.mx (L.C.-S.); javierp@unam.mx (J.P.); 4 Departamento de Bioquímica. Instituto Nacional de Cardiología “Ignacio Chávez”. Juan Badiano 1. Tlalpan 14080, Cd. de México, Mexico; mohammed.elhafidi@cardiologia.org.mx

**Keywords:** fumonisin B1, *Fusarium verticillioides*, H^+^-ATPase, long chain bases, maize embryos, plant plasma membrane, sphingolipids

## Abstract

Fumonisin B1 is a mycotoxin produced by *Fusarium verticillioides* that modifies the membrane properties from animal cells and inhibits complex sphingolipids synthesis through the inhibition of ceramide synthase. The aim of this work was to determine the effect of Fumonisin B1 on the plant plasma membrane when the mycotoxin was added to germinating maize embryos. Fumonisin B1 addition to the embryos diminished plasma membrane fluidity, increased electrolyte leakage, caused a 7-fold increase of sphinganine and a small decrease in glucosylceramide in the plasma membrane, without affecting phytosphingosine levels or fatty acid composition. A 20%–30% inhibition of the plasma membrane H^+^-ATPase activity was observed when embryos were germinated in the presence of the mycotoxin. Such inhibition was only associated to the decrease in glucosylceramide and the addition of exogenous ceramide to the embryos relieved the inhibition of Fumonisin B1. These results indicate that exposure of the maize embryos for 24 h to Fumonisin B1 allowed the mycotoxin to target ceramide synthase at the endoplasmic reticulum, eliciting an imbalance of endogenous sphingolipids. The latter disrupted membrane properties and inhibited the plasma membrane H^+^-ATPase activity. Altogether, these results illustrate the mode of action of the pathogen and a plant defense strategy.

## 1. Introduction

The plant plasma membrane (PM) is the boundary of the cell and constitutes an important barrier against both pathogenic microorganisms and environmental stresses. Changes in the PM properties during fungal infection may be induced by secondary metabolites, such as toxins synthesized by the pathogen. Microbial toxins, for instance syringomicin, fusiccocin, beticolins, and fungal elicitors modify some properties and enzymatic activities of the host PM [1,2,3]. Fumonisin B1 (FB1) is a mycotoxin produced by several *Fusarium* spp. Among those, *Fusarium verticillioides* is the major ear rot fungus of corn and an important contaminant of stored grains worldwide [4]. FB1 inhibits radicle elongation and amylase production in germinating seeds [5]. In animals, FB1 produces equine leucoencephalomalacia, porcine pulmonary edema, and rodent hepatic cancer among other toxic effects [6,7]. Consumption of *F. verticillioides* contaminated corn has been correlated with an increased incidence of human esophageal cancer in Southern Africa and China [8,9,10,11,12,13,14]. Three molecular targets of the FB1 have been described in plants so far: Ceramide synthase (CS) [15], low pHi α-amylase isoforms [5], and the PM H^+^-ATPase [16].

FB1 is the diester of propane-1,2,3-tricarboxylic acid and 2-amino-12,16-dimethyl, 3,5,10,14,15-pentahydroxyicosane with both C-14 and C-15 hydroxy groups esterified to the terminal carboxy groups of the acids [6]. It interacts with lipid bilayers as experiments with liposomes and Langmuir films have shown that FB1 perturbs membrane order and increases lipid peroxidation [17,18]. We have determined that FB1, when directly added to isolated PM increases the fluidity in the hydrophobic region of the bilayer and inhibits the PM H^+^-ATPase [16]. This H^+^ pump is a key enzyme in the plant cell physiology, since it produces a transmembrane H^+^ gradient which drives secondary transport of solutes for cell nutrition, promotes cell elongation, and stomata opening [19,20,21].

It is well established that FB1 disrupts the biosynthesis of sphingolipids by inhibiting CS, therefore increasing the levels of precursor long chain bases (LCBs) and decreasing ceramide, the product of the reaction, in both plant [15,22,23] and animal [24] cells.

In this work, we found that when maize embryos were germinated in the presence of FB1, PM sphinganine levels increased dramatically, while glucosylceramide slightly decreased, such changes produced a PM with increased permeability and decreased fluidity. Moreover, a 30% inhibition of the PM H^+^-ATPase was observed, which was not associated to the raise in sphinganine levels but to complex sphingolipids diminution as the addition of ceramide relieved FB1 inhibition.

## 2. Results

### 2.1. FB1 Addition to the Maize Embryos Inhibits the PM H^+^-ATPase Activity

**Figure 1:**
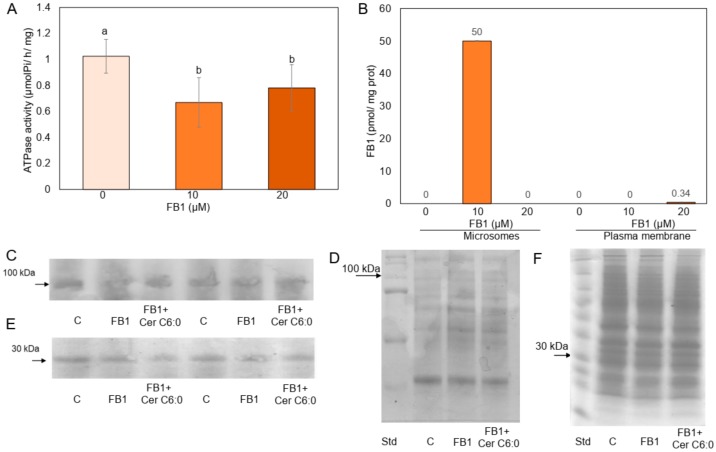
Effect of Fumonisin B1 (FB1) on the plasma membrane (PM) H^+^-ATPase activity, mycotoxin content, and H^+^-ATPase and 14-3-3 protein levels in the PM. Maize embryos were imbibed with or without FB1 at the indicated concentrations during 24 h. Then, microsomes or PM were isolated and ATPase activity, FB1 or H^+^-ATPase, and 14-3-3 protein levels were determined. (**A**) Five to 10 µg of PM protein were added to a reaction mixture to measure ATP hydrolysis in the absence or presence of FB1^.^ (**B**) FB1 was determined in microsomes or PM as described under Experimental Procedures. The amount of mycotoxin in microsomes was of 50 ± 17 pmol/mg protein when embryos were exposed to 10 µM FB1 and of 0.34 ± 0.03 pmol/mg protein in the PM when embryos were exposed to 20 µM FB1. No mycotoxin was detected in both membranes when the embryos were exposed at the other concentrations shown. Data are the means ± SD of three independent experiments with three different membrane preparations. Welch’s *t*-test and two-way ANOVA followed by Bonferroni’s multiple comparisons test were performed using GraphPad Prism version 8.0 for Windows, GraphPad Software (San Diego CA). (**C**) Effect of FB1 on the PM levels of H^+^-ATPase. Twenty-five µg of PM protein from maize embryos imbibed in the absence or presence of 10 µM FB1 or to 10 µM FB1 and Ceramide 6:0 as indicated, were subjected to western blot for PM H^+^-ATPase detection. (**D**) Replicate samples in the same gels were Coomassie blue stained. Experiment is representative of 10 experiments carried out with 10 independent membrane preparations exposed to FB1 or eight exposed to Ceramide 6:0. Density recordings of the bands were done and the values analyzed by the ANOVA test (*P* = 0.8 > α = 0.05). (**E**) Effect of FB1 on the PM levels of 14-3-3 protein. Twenty-five µg of PM protein from maize embryos imbibed in the absence or presence of 10 µM FB1 or to 10 µM FB1 and Ceramide 6:0 as indicated, were subjected to western blot for 14-3-3 protein detection. (**F**) Replicate samples in the same gels were Coomassie blue stained. The experiment is representative of 16 experiments carried out with 10 independent membrane preparations exposed to FB1 or 10 exposed to Ceramide 6:0. Density recordings of the bands were done and the values analyzed by the ANOVA test (*P* = 0.1 > α = 0.05). Cer C6:0: Ceramide C6:0.

In order to investigate whether FB1 could reach intracellular targets that affected the PM, the mycotoxin was added to the maize embryos and then the isolated PM vesicles were studied. Figure 1A shows that the H^+^-ATPase activity from PM vesicles isolated from maize embryos exposed to FB1 was inhibited 35% and 24% with 10 and 20 µM FB1, respectively. Since FB1 inhibits the H^+^-ATPase activity from PM in vitro at similar extent in an uncompetitive mechanism [16], we tested the possibility that FB1 present in the membrane was responsible of this inhibition, therefore, measurements of FB1 levels in the isolated PM and microsomal fractions were carried out and the results are shown in Figure 1B. Microsomes isolated from embryos exposed to the lower mycotoxin concentration contained low levels of FB1, but the mycotoxin was not detected in the PM exposed to 10 µM FB1 and only traces were found in the vesicles when the embryos were exposed to 20 µM FB1. These results indicated that the in vivo activity of FB1 was not related to its presence in the membrane and therefore suggested that the mycotoxin effect was not due to a direct interaction with the PM enzyme but to a FB1 inhibition on CS, an ER located enzyme, as previously reported [15,24]. Most of all, these results indicated that the H^+^-ATPase inhibition observed when 10 µM FB1 was added to the maize embryos was not associated to some FB1 remaining in the membrane. In order to test the possibility that FB1 could be inhibiting the synthesis of the H^+^-ATPase or its incorporation to the PM, the enzyme was immunodetected. Figure 1C,D shows that the amount of the enzyme was unchanged in the membrane after embryos exposure to 10 µM FB1. Since protein 14-3-3 associates to the phosphorylated and active form of the H^+^-ATPase, the possibility that 14-3-3 proteins were in minor amounts in the PM from embryos exposed to 10 µM FB1 was explored. The results in Figure 1E,F show that this was not the case, as PM from control and FB1-exposed embryos had the same amount of 14-3-3 proteins. In order to assure that protein synthesis was not disrupted by the mycotoxin [25], the amount of total protein was determined in microsomes and PM from the control and 10 µM FB1-treated embryos. Table S1 shows that the amount of total protein was the same irrespective of whether or not the embryos were exposed to FB1.

### 2.2. Sphingolipid Species Vary in the PM from Maize Embryos Exposed to FB1

**Figure 2:**
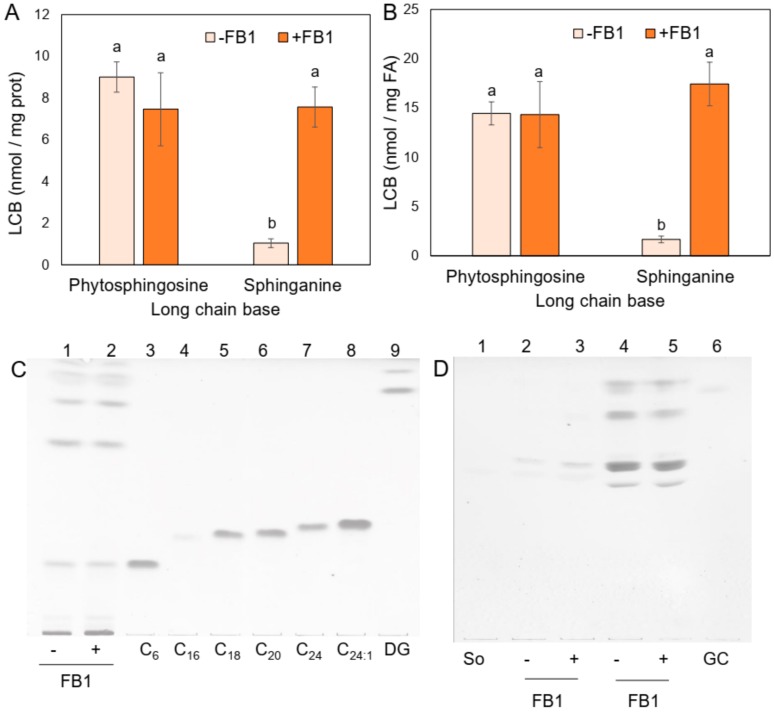
Levels of long chain bases (LCBs), ceramides, and glucosylceramide in PM from maize embryos exposed to FB1. Maize embryos were exposed to 10 µM FB1 for 24 h and PM fractions were isolated, lipid extracts obtained and LCBs, ceramides and glucosylceramide determined. (**A**) LCBs determined are expressed in terms of nmol/min/mg protein. (**B**) LCBs determined were expressed in terms of nmol/min/mg fatty acid. Data are the means ± SD of six independent experiments with at least three different membrane preparations. Holm–Sidak *t*-test was performed using GraphPad Prism version 8.0 for Windows, GraphPad Software (San Diego, CA, USA). (**C**) Ceramides were determined in extracts containing the equivalent to 18 µg of PM protein and then separated in thin layer chromatography (TLC) plates eluted with a mobile phase of CHCl_3_:CH_3_OH:CH_3_COOH, 94:1:5 v:v:v and developed with a copper solution. Lanes 1 and 2 were loaded with 18 µg of PM extract from control and 10 µM FB1-imbibed embryos. Lanes 3–8 contained 20 nmol of the indicated ceramides and lane 9 contained 20 nmol of a diglyceride (DG) with C18:0 and C20:0 fatty acids. (**D**) Glucosylceramides were detected in extracts containing the equivalent to 18–36 µg of PM protein and then separated in TLC plates eluted with a mobile phase of CHCl_3_:CH_3_OH:CaCl_2_0.22 %, 60:35:8 *v:v:v* and developed with a copper solution. Lane 1 contained 10 nmol of sphingosine, lanes 2 and 3 were loaded with 18 µg of PM extract from control and 10 µM FB1-imbibed embryos, lanes 4 and 5 were loaded with 36 µg of the same extracts and lane 6 contained 20 nmol of glucosylceramide. Data are representative of at least four independent experiments with at least three different membrane preparations. DG: Diglyceride with C18:0 and C20:0 fatty acids; C_6_: Ceramide C6:0; C_16_: Ceramide C16:0; C_18_: Ceramide C18:0; C_20_: Ceramide C20:0; C_24_: Ceramide C24:0; C_24:1_: Ceramide C24:1; GC: Glucosylceramide; So: Sphingosine.

As a result of CS inhibition by FB1, LCBs are not acylated and therefore accumulate while the amount of complex sphingolipids decrease [15,24]. Levels of endogenous free LCBs are very low in plants [23] and hence an extraction of total LCBs (free) and after alkaline hydrolysis (from complex sphingolipids) was performed in order to measure a possible difference of endogenous LCBs in the PM upon FB1 embryo-treatment. Figure 2A,B shows that while phytosphingosine levels were similar, sphinganine content increased more than 7.3-fold (expressed as nmol/mg prot or 10-fold in nmol/mg fatty acid) in the PM from FB1-treated embryos as compared to the control embryos.

Detection of free ceramides by TLC was unsuccessful in the FB1 treated or nontreated embryos (Figure 2C) but glucosylceramide levels slightly diminished (Figure 2D). These results were consistent with the expected action of FB1 on CS and the effect of the mycotoxin observed in *Arabidopsis thaliana* [23]. Therefore, the FB1-treated embryos produced a PM with a high sphinganine content and a decrease of glucosylceramides. Such changes could be associated to the inhibition of the H^+^-ATPase activity.

### 2.3. Changes in Sphingolipids as a Result of FB1 Addition to the Maize Embryos are Associated to an Increase in the Permeability and a Decrease in the Fluidity of the PM

**Figure 3:**
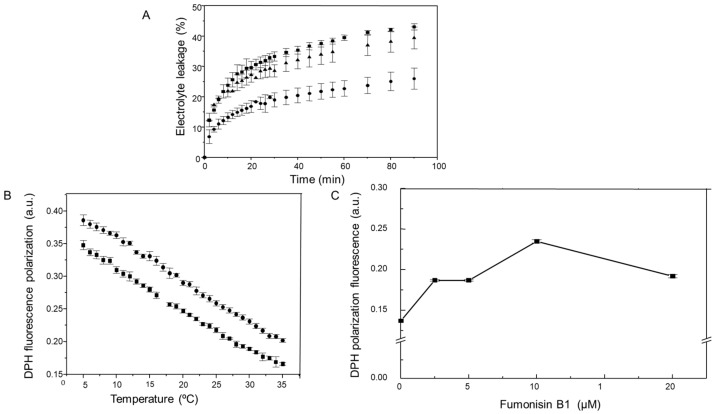
Effect of FB1 on permeability and fluidity of PM from maize embryos. (**A**) Effect of FB1 on maize embryo permeability. Twenty embryos were imbibed in 0 (●), 10 (▲), 20 (■) µM FB1, then transferred to deionized water and electric conductivity measured as described under Experimental Procedures. Data represent means ± SD from 5–10 independent assays with at least three different embryo batches. (**B**) Effect of FB1 on the PM fluidity from maize embryos. Fluidity was measured in the PM isolated from maize embryos imbibed in the absence (■), or presence of 10 µM FB1 (●) or (**C**) at the indicated FB1 concentrations for 24 h. Fluidity was measured by 1,6-diphenyl- 1,3,5-hexatriene (DPH) fluorescence polarization from 5 to 35 °C (**B**) or at 30 °C (**C**). Data represent means ± SD of at least three experiments done with a minimum of three different PM preparations.

The observed increase of endogenous sphingolipid species in the PM could be affecting the bilayer properties and these changes, in turn, could be contributing to the observed PM H^+^-ATPase activity inhibition. Therefore, we measured the permeability levels of the maize embryos exposed to FB1 and the fluidity of the PM isolated from FB1-imbibed embryos. Figure 3A shows that 10 and 20 µM FB1 increased more than 2-fold the maize embryos nonspecific electrolyte loss. DPH fluorescence polarization determined as a function of temperature demonstrated that PM fluidity from maize embryos imbibed with FB1 was decreased relative to the control membranes, in the range of temperatures assayed (Figure 3B). We also tested the effect of different FB1 concentrations in the maize embryos imbibition medium on the PM fluidity, determining DPH fluorescence polarization at 30 °C, the same temperature at which ATPase activity was measured. Results in Figure 3C show that membrane fluidity considerably diminished upon FB1 treatment and that the largest effect was with 10 µM FB1. Higher mycotoxin concentrations did not increase fluidity proportionally.

### 2.4. FB1 Addition Does Not Modify the Profile of the PM Main Fatty Acids

**Figure 4:**
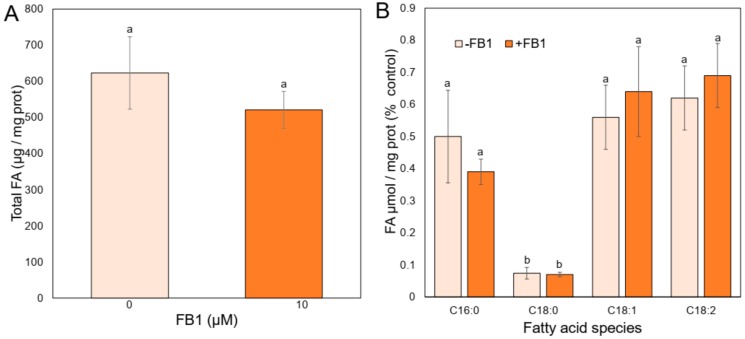
Fatty acids from PM of maize embryos exposed to FB1. Maize embryos were imbibed with 10 µM FB1 for 24 h, their PM fractions isolated, membrane lipids extracted and derivatized to obtain the correspondent methyl esters to be analyzed by gas chromatography. (**A**) Total fatty acids from PM from control and FB1-imbibed maize embryos. (**B**) Main fatty acids from control and FB1-imbibed maize embryos. Data are the means ± SD of 3–5 independent experiments. Holm–Sidak *t*-test was performed using GraphPad Prism version 8.0 for Windows, GraphPad Software (San Diego, CA, USA).

According to the fluidity results, an effect of the mycotoxin addition on the fatty acid content of the PM was explored, as it is known that membrane fatty acids composition is one of the major factors influencing membrane fluidity. We measured the levels of the more abundant fatty acids of the PM from maize embryos [26] when these were imbibed with or without FB1. Figure 4A shows that the total amount of fatty acids was unaffected in the PM from the embryos treated with FB1 as compared to the control embryos. The main species of fatty acids identified in every condition were: Palmitic (C16:0), stearic (C18:0), oleic (C18:1), and linoleic (C18:2) acids (Figure 4B). Their levels remained unchanged in the PM from maize embryos imbibed with FB1 as compared to the control membranes. According to the presence of the double bonds, the ratio of the saturated fatty acids to unsaturated fatty acids was the same as well. Therefore, the bond index stayed unvaried in the presence of the mycotoxin, and FB1 at 10 µM did not alter the fatty acid/protein ratio in PM, according to the absence of disparity on the amount of total protein in the PM in both treatment conditions (Table S1). These results indicated that changes in fluidity and permeability could not be assigned to the composition of the main membrane fatty acids. 

### 2.5. LCBs but Not Ceramide Inhibit H^+^-ATPase Activity When Directly Added to the PM

**Figure 5:**
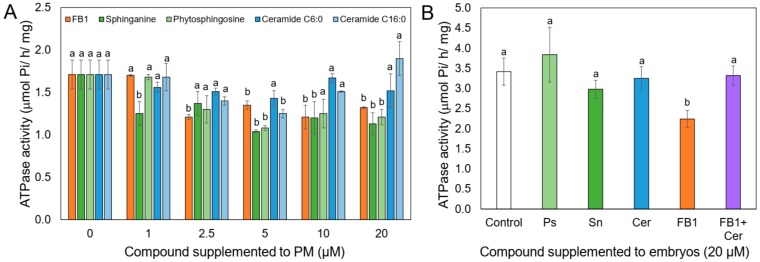
Effects of FB1, sphinganine, phytosphingosine, Ceramide C6:0 and C16:0 on the H^+^-ATPase activity when added to the isolated PM or to the maize embryos. (**A**) Maize embryos were imbibed in the absence of FB1 for 24 h, then PM were purified and exposed to each compound at the indicated concentrations. Five to 10 µg of membrane protein were used to assay ATP hydrolysis. Means and SE (n = 3) of enzymatic determinations are expressed, followed by letters indicating whether the concentration of each compound had a significant (*P* < 0.05) influence on ATPase activity as determined by the t-student comparison of means. (**B**) Maize embryos were exposed to 20 µM of every sphingoid compound or FB1 for 24 h. Then, PM vesicles were isolated and ATP hydrolysis measured as phosphate release as described under Materials and Methods. Data are means of three independent experiments ± SD. Two-way ANOVA followed by Bonferroni’s multiple comparisons test and one sample *t*-test were performed using GraphPad Prism version 8.0 for Windows, GraphPad Software (San Diego, CA, USA). Cer: Ceramide 6:0; Ps: Phytosphingosine; Sn: Sphinganine.

FB1 has an inhibitory effect on the PM H^+^-ATPase when directly added to isolated PM [16]. Given the structural similarity of sphinganine with the mycotoxin and the high levels of sphinganine in the PM as a consequence of FB1 addition, we assayed the in vitro effect of sphinganine and of phytosphingosine as well, on the H^+^-ATPase activity. Figure 5A shows that both, LCBs and FB1, inhibited ATPase activity at about the same extent, i.e., 20% at 10 and 20 µM mycotoxin concentration. In contrast, neither Ceramide C6:0 and C16:0 had an inhibitory effect on the H^+^-ATPase activity. Taken together, these results suggested that the accumulation of endogenous sphinganine or the glucosylceramide decrease in the PM after the mycotoxin addition to the maize embryos could explain the H^+^-ATPase inhibition observed.

### 2.6. Ceramide Releases the FB1 Inhibition on the PM H^+^-ATPase Activity, When Both Compounds are Added Together to the Maize Embryos

Since FB1 inhibition on CS activity produces an increase of endogenous LCB levels that is accompanied by a decrease of ceramides, the product of the reaction of LCBs acylation, it was necessary to explore whether a deficit of this compound could be affecting the H^+^-ATPase activity. The rationale behind this experiment was that if a lessening in the endogenous ceramide driven by the FB1 addition was responsible to some extent to the H^+^-ATPase inhibition observed, the simultaneous supplementation of FB1 and ceramide to the maize embryos could result in a correspondent recovery of the activity. The results shown in Figure 5B indicated that this was the case. Whereas FB1 produced the 30% inhibition previously observed, simultaneous addition of FB1 and ceramide restored the activity to the control values. Surprisingly, when sphinganine, phytosphingosine, or ceramide alone were added to the embryos, H^+^-ATPase activity was unaffected with respect to the control, in contrast to the inhibitory action of those LCBs when directly added to the isolated PM (Figure 5A). This finding indicated that a decline in the endogenous ceramide levels was responsible for the in vivo H^+^-ATPase inhibition observed in maize embryos germinating in the presence of FB1. When H^+^-ATPase levels were estimated by western blot in the PM isolated from the embryos exposed to FB1 and ceramide, levels were equal to the control or FB1-treated embryos (Figure 1C,D). The same was found for the levels of 14-3-3 proteins (Figure 1E,F). 

## 3. Discussion

The present work brings insights on the effects of the fungal toxin FB1 on the plant PM: How the lipid bilayer is affected, how the PM H^+^-ATPase activity is decreased and how these effects can be envisioned in terms of the interaction of the pathogen with the plant cell. Noteworthy, this work provides experimental evidence that ceramides/complex sphingolipids from PM influence H^+^-ATPase activity from plants. 

Our system of maize embryos exposed to the mycotoxin allows to study its effects on cell components beyond the PM, since at 10 µM FB1 and 24 h of imbibition, the mycotoxin was no longer detected in this membrane, but could still influence the structure and function of the bilayer. This is an important fact to be considered, given the pleiotropic effects that FB1 has shown in all the living systems tested [6,15,16,27,28,29]. Due to the temporality of the FB1 action, previous experiments carried out with FB1 directly added to the PM (in vitro) can be interpreted in terms of the effects that can take place at the early times of exposure of the plant cell to the mycotoxin/fungus [16]. Our results in the present work show that providing longer times elapse, the fungal toxin can reach intracellular targets that have a repercussion in the PM structure and function. 

### 3.1. Effects of FB1 on the Lipid Bilayer of the PM from Maize Embryos

Accumulation of LCBs occurs as a consequence of FB1 inhibition on the CS in plants [15,22,23], yeast [27], and animal cells [30]. We found a sphinganine increase in the PM upon in vivo FB1 addition to the maize embryos. This was associated to a decrease in fluidity and an increase in PM permeability. Such effects are consistent with the structure of sphinganine, which has a small polar head group that can form hydrogen bond networks as those reported for ceramides [31], which contribute to induce membrane rigidity and could possibly form regions in the PM with decreased acyl motion. This effect originates lateral phase separation leading to an increase in membrane permeability, as described in model and erythrocyte membranes for sphingosine (a LCB with a double bond in the C4 of the sphinganine structure) [32]. The enhanced permeability could also be related to a sphinganine effect of pore formation in the PM, as reported for sphingosine as well [33]. Host-nonselective toxins such as FB1 often cause an increase in cellular ions and water loss [2,3]. The maize embryos imbibed with FB1 showed an increase in electrolyte leakage that decreased at concentrations of the mycotoxin ≥ 10 µM. These results contrast with those obtained with *Datura stramonium* leaves treated with FB1 (1–100 µM), where the electrolyte leakage increased proportionally to the mycotoxin concentration [34].

We were unable to detect a concomitant decrease in the content of free ceramides and only a small descent in complex sphingolipids from the PM from FB1-treated embryos. This was probably due to the lack of sensitivity of the TLC procedure or to the possibility that maize embryos at 24 h of imbibition showed a low sphingolipid turnover. The latter could be explained by the limited sphingolipid synthesis rate and a small degradation rate of pre-existing complex sphingolipids. If a substantial decrease of ceramide/complex sphingolipids had occurred upon FB1 addition, an increase in membrane fluidity should have been observed, which was not the case. However, it is convenient to determine complex sphingolipids such as ceramides, glucosylceramides, and glycosylinositolphosphoceramides in the PM with a more sensitive technique upon exposure of embryos to FB1. 

Changes in fluidity produced by FB1 addition were not related to the levels of major fatty acids in the membrane. Rather, our results on the PM H^+^-ATPase and the action of FB1 on CS [15,23,24] indicate that fluidity was altered by an increase in LCBs on the basis of their ordering effect on membrane structure, increasing membrane rigidity [35]. 

### 3.2. Effect of FB1 on the PM H^+^-ATPase Activity from Maize Embryos

Our experiments testing FB1 effects on the maize embryos revealed that PM activity of the H^+^-ATPase was partially dependent on endogenous ceramide levels, because the addition of ceramide together with FB1 overcame the inhibitory effect of the mycotoxin on the PM H^+^-ATPase activity. This implied that in our system, 24 h of imbibition were enough for embryo cells to incorporate exogenous ceramide. 

FB1 added directly to isolated PM exerts an uncompetitive inhibition on the plant PM H^+^-ATPase [16]; however, in the present work, this effect is very unlikely to occur, as no traces of FB1 were found in the membrane. In addition, the inhibitory action of the mycotoxin could not be explained as an effect on protein synthesis as it has been previously described for monkey kidney cells [25] or to a mistargeting of the enzyme to the vacuole as a result of an impairment in sphingolipid synthesis (as it has been found for the Pma1p H^+^-ATPase isoform from yeast [36], because the same amount of total protein was recovered in both preparations (Table S1) and equal levels of H^+^-ATPase were immunodetected in PM from embryos exposed to the mycotoxin and in the control. Neither the H^+^-ATPase inhibition could be related to a different association extent of the 14-3-3-protein, a H^+^-ATPase regulatory protein, since levels of this protein were unaffected in the PM preparations from embryos exposed to FB1 as compared to the control. Nor the decrease in PM fluidity upon sphinganine accumulation provides an explanation of the ATPase activity inhibition, since we and other groups have proved that changes in membrane fluidity do not influence this enzyme activity in maize [16,37]. This work also showed that the abundance of sphinganine in the plasma membrane generated by FB1 addition was not the cause of the H^+^-ATPase inhibition, as it was indicated by the experiments wherein exogenous sphinganine was added in vivo and no effect on the H^+^-ATPase activity was found. It could be argued that incorporation of this exogenous compound to the membrane was inefficient, but it has been reported that LCBs are readily taken up into the membranes as compared to ceramides, which show slower uptake kinetics [38] but that in our conditions, was incorporated into the maize embryos. It was possible that sphinganine added to the embryos was acylated and converted into ceramide, which is unable of inhibiting the H^+^-ATPase activity (according to in vitro experiments). This case is not plausible, since when FB1 was added together with ceramide to the embryos, endogenous levels of sphinganine should have been increased, inhibiting the ATPase activity as expected with FB1 alone. However, the presence of ceramide (together with FB1), released such inhibition even in the presence of high sphinganine levels in the membrane. 

Free ceramide or ceramide as part of complex sphingolipids such as glucosylceramides influence the H^+^-ATPase activity through two different ways: As an immediate neighbour membrane lipid of the enzyme or as an effector of specific regulatory components of the enzyme, namely proteins or other lipids [16,39]. A third possibility combining these two aspects could be conceivable as well. In any case, either free ceramide or ceramide as moiety of complex sphingolipids might be responsible for these effects. 

Additional evidence supporting the first option includes the finding that the PM H^+^-ATPase resides in DRM isolated from yeast [40] and plant [41,42,43] PM. The components of DRM are sphingolipids, sterols, and some phosphoglycerolipids, whose structure favours tight intermolecular packing [44]. PM showing the presence of DRM enriched in H^+^-ATPase in maize embryos confirms the association of the enzyme with this kind of lipids [45] and suggests that ceramide, either free or as the backbone of membrane complex sphingolipids, is in close contact with the PM H^+^-ATPase and is required for its activity, conferring an adequate lipid microenvironment for optimal catalytic motion [46], or even for stability, as it has been demonstrated for Pma1-10 from yeast [47]. This possibility can also be supported by a large body of experimental evidence that shows that sterols are required for the activity of the H^+^-ATPase [37,48,49], as these lipids are in close association with ceramide-containing lipids in membrane microdomains [41,50,51,52] and that maize PM has a high sterols content [53].

An alternative interpretation for our data is that ceramide or one of its phosphorylated derivatives interacts with a PM H^+^-ATPase regulatory protein, such as a phosphatase. Low activity states of the PM H^+^-ATPase correlate with a hypophosphorylated form of the enzyme [54] and a decrease in the association of 14-3-3 proteins [55], which have been consistently detected in plant PM microdomains [41,42,45]. However, as mentioned above, changes in 14-3-3 protein levels were not found in our conditions. Nevertheless, the possibility that endogenous free ceramide, complex sphingolipids, or their phospho-derivatives could be activating some dephosphosphorylating system acting on the H^+^-ATPase cannot be discarded, since, as it has been described, sphingolipids as sphingosine-1P or phytosphingosine-1P are mediators in signal transduction pathways in plant cells [56,57], similarly to what happens in animal systems [38,58]. This view is supported by the vast diversity and abundance of sphingolipids in plants [59,60].

It is important to notice that when PM were incubated with sphinganine or phytosphingosine, the H^+^-ATPase activity was inhibited. In this condition, where no sphingolipid metabolism occurs, LCBs added could freely interact with the PM. Thus, the observed H^+^-ATPase inhibition suggests that LCBs could be occupying in the H^+^-ATPase the same site in which FB1 exerts the uncompetitive inhibition. This is possible, given the resembling structure of these molecules. In fact, this is the reason why FB1 and LCBs are recognized by the catalytic site of the CS [61]. Our data, together with the postulation that the activity of PM H^+^-ATPase works as a switch for plant defense responses must be considered [54,62,63]. In this context, whether ceramide requirement for PM H^+^-ATPase activity is related to a pattern of defense response against pathogens in which membrane microdomains are involved, remains to be established, but it is worth mentioning that DRM, structures in which the H^+^-ATPase is abundant, clearly contain distinctive elements of defense reactions in plants [41,42,43,63]. 

### 3.3. Relevance of the Effects of FB1 in the Plant–Pathogen Interaction

A differential accumulation of LCBs in maize genotypes depending on their resistance to *F. verticillioides* has been reported. Maize resistant hybrids show increased sphinganine levels while susceptible hybrids accumulate phytosphingosine after infection with *F. verticillioides* [64]. It is known that part of the LCBs that are accumulated under FB1 addition display a signaling role that leads to a programmed cell death in the hypersensitive response (HR) [23], the most effective defense process of plants against pathogens. It is tempting to speculate that the excess of LCBs contributes to the loss of membrane structure and permeability produced during HR. 

Altogether, these results indicate that FB1, recognized as a virulence factor that is secreted by *Fusarium verticillioides* targets the plant PM, modifying its composition, fluidity, permeability, and the activity of the most important primary pump of the PM. These effects are produced through a direct action of the mycotoxin on the membrane, evidenced by in vitro studies [16] and through an interaction of the mycotoxin with the de novo synthesis of sphingolipids, illustrated by in vivo studies (this work). The inhibition of the PM H^+^-ATPase produced both by the FB1 molecule itself [16], and by an elicited ceramide/complex sphingolipid deficiency is consistent with the decline of radicle elongation and medium acidification observed by in vivo treatment with FB1 [5,16]. The kinetics of mycotoxin production by the pathogen and of the mycotoxin uptake by the plant cell would be determining the extent of the FB1 deleterious effects on the PM in vivo.

## 4. Materials and Methods

FB1, sphinganine, phytosphingosine, and *N*-Hexanoyl-D-*Erythro*-sphingosine (C6:0 ceramide), were purchased from Sigma-Aldrich (St. Louis, MO, USA) and *N*-Palmitoyl-D-*Erythro*-sphingosine (C16:0 ceramide) from ICN (Irvine, CA, USA). Optiprep and 1,6 diphenyl-1,3,5-hexatriene (DPH) were obtained from Sigma-Aldrich (St. Louis, MO, USA). Triton X-100 was purchased from Pierce (Rockford, Ill). Antibody against beet PM H^+^-ATPase was a kind gift from Dr. Luis E. González de la Vara (CINVESTAV, Irapuato, Mexico). Antibody against 14-3-3 β isoform was obtained from Santa Cruz Biotechnology Inc. (Santa Cruz, CA, USA). Alkaline-phosphatase conjugated goat anti-rabbit IgG was obtained from Sigma-Aldrich (St. Louis, MO, USA). Uridine diphosphate glucose, [glucose-1-^3^H] was purchased from NEN Life Sciences Products, Inc. (Boston, MA, USA). All the other chemicals were of the highest purity available.

### 4.1. Imbibition of Maize Embryos

Maize (*Zea mays*, landrace Chalqueño) embryos were manually dissected from dry seeds, imbibed, and supplemented with FB1 dissolved in H_2_O or with sphinganine, phytosphingosine, or ceramide dissolved in ethanol and incubated at 29 °C for 24 h as previously described [16].

### 4.2. Measurement of Electrolyte Leakage

Maize embryos (20 embryos) were imbibed with or without FB1 for 24 h and then were transferred to deionized water, where electrical conductivity was measured with a conductimeter CONMET1 (Hanna Instruments, Padova, Italy) for 1–1.5 h at 25 °C and compared to the conductivity of deionized water. When the measurement was finished, the sample of water and embryos was boiled for 10 min, cooled to 25 °C, and the electric conductivity was determined and considered as total electrolytes of the sample in order to calculate the percent of electrolytes released into the deionized water, during the period of measurement.

### 4.3. Isolation of PM Vesicles

Embryos imbibed for 24 h were frozen and homogenized as described previously [16] and then PM were isolated by the procedure in [65] until the obtention of the U_2_ fraction. The membrane suspension had an ATPase activity that was 4–fold enriched in glucan synthase II activity (a PM enzyme marker) and was 75%–85% vanadate-sensitive. This PM fraction was aliquoted and stored at –70 °C.

### 4.4. Determination of ATP Hydrolysis

This was done in the conditions used previously [16], quantitating phosphate release from ATP as described [66]. Phosphate release was linear up to 2 h of incubation, both in the presence and absence of FB1 or the sphingolipids. Blanks to estimate nonenzymatic ATP hydrolysis were included. Preincubation of the PM vesicles with FB1 or the sphingolipids did not modify the effect on ATPase activity (Figure S1). The amounts of ATP and MgCl_2_ necessary to obtain the desired concentration of substrate-metal complex and free Mg^2+^ were calculated as described [67]. The predominant complex in our conditions was MgHATP^-^, whose concentration was 8.11 mM while keeping free Mg^2+^ at 35 µM.

### 4.5. Measurement of PM Fluidity

PM fluidity was measured by fluorescence polarization of 1,6 diphenyl-1,3,5- hexatriene (DPH) as reported [16].

### 4.6. Determination of FB1

Extraction of FB1 was carried out from PM vesicles (300–500 µg of membrane protein in 200 µL) isolated from control or FB1-imbibed maize embryos and supplemented with 1 mL of acetonitrile:water (60:40, *v:v*) and vortexed for 15 min. This mixture was centrifuged at 12,000 rpm during 15 min in a microcentrifuge (Sorvall, MC12, Newtown, CT), the supernatant was transferred to a vial and evaporated with hot air under nitrogen flow to dryness. The residue was resuspended in 100 µL of acetonitrile/water (1:1, *v:v*), supplemented with 400 µL of 1% KCl, mixed and loaded into a solid–phase extraction cartridge (Sep-Pak C_18_, Waters/Millipore, Milford, MA), that was previously conditioned with 2 mL of methanol and 2 mL of 1% KCl (*w:v*). After the sample passed through the column, it was washed with 2 mL of 1% KCl and then with 2 mL of acetonitrile/water (1:5, *v:v*). FB1 was eluted with 2 mL of acetonitrile/water (7:3, *v:v*), collected and evaporated to dryness. The residue was resuspended in 100 µL of acetonitrile/water (1:1, *v:v*). FB1 recovery was assessed by adding 4 nmol of FB1 to control membranes at the start of the procedure and then determined after the extraction procedure was followed. Quantitation of FB1 was done by high performance liquid chromatography (HPLC) essentially based on the method previously reported [68]. Briefly, the separation of the samples was conducted using a SuperCosil LC-18-Si reverse-phase column (15 cm × 4.6 mm, particle size 5 µm (Supelco, St. Louis, MO, USA) and an isocratic solvent system (methanol:NaH_2_PO_4_ 100 mM, pH 3.3, at 68/32, *v:v*, with phosphoric acid). Ten µl from the sample or from the FB1 standard solution (1 mM dissolved in acetonitrile/water 1:1, *v:v*) were reacted with 5 µL of *o*-phthaldialdehyde (OPA) reagent (2.5 mg OPA dissolved in 50 µL of ethanol, 2.45 mL of 3% boric acid, pH 10.5 and 2.5 µL of 2-mercaptoethanol) and diluted with 190 µL of mobile phase without phosphoric acid and 50 µL of 5 mM K_2_HPO_4_. It was incubated 1 min and then injected to a Shimadzu chromatograph (model LC-10AD (Shimadzu Corporation, Kyoto, Japan) with a fluorescence detector, using 335 and 440 nm as excitation and emission wavelengths, respectively. A standard curve of FB1 dissolved in acetonitrile/water (1:1, *v:v*) in the range from 0.0166 to 0.0667 pmol was done.

### 4.7. Measurement of LCBs from PM

To extract total LCBs (free and as a moiety of complex sphingolipids), 100 µg of microsomal or PM protein were supplemented with 1.5 mL of a solution containing 9 mM KCl, 19 mM KOH, and the mixture was extracted with 4 mL of ethyl acetate by gentle rotation for 40 min. The phases were separated by centrifugation at 1000× *g* for 15 min. The organic phase was recovered and evaporated to complete dryness at room temperature under N_2_ [69]. The residue obtained was dissolved in 200 µL of absolute ethanol. An aliquot of 1–20 µL was taken for OPA derivatization. This was done by mixing OPA reagent (2.5 mg OPA, 50 µL of ethanol, 2.5 µL of β-mercaptoethanol and 3% boric acid solution adjusted to pH 10.5 with KOH up to a final volume of 1.5 mL). An aliquot of 1–20 µL of lipid residue resuspended in ethanol and 50 µL of the OPA reagent was added to the mobile phase (methanol/5 mM phosphate buffer pH 7.8, at 89/11, *v/v*) to complete a total volume of 500 µL. The mixture was stirred 2 min under dark conditions, sonicated for 12 min and maintained in the bath for 18 min more. Derivatized samples were kept on ice for 30 min before LCBs separation by HPLC (model LC-10AD, Shimadzu Corporation, Kyoto, Japan) using a C_18_ column (15 cm × 4.6 mm, 5 µm, Supelco, St. Louis, MO, USA). The derivatives were quantified by fluorescence detection (excitation and emission wavelengths of 337 and 448 nm, respectively), at a flow rate of 1.3 mL/min. Six hundred pmol of sphingosine (a mixture of threo- and erythro-isomers of 59% and 39%, respectively) as internal standards were also added.

### 4.8. Measurement of Fatty Acids from PM

One to 2 mg of PM protein were supplemented with 50 µl of *L*-α-diheptodecanoyl phosphatidylcholine (1 mg/mL) as internal standard. Then, one ml CH_3_OH, 500 µL of 0.9% NaCl (*w:v*) and 2 mL CHCl_3,_ were added to PM, vortexed 30 s and centrifuged for 2 min at 5000 rpm. A second CHCl_3_ (2 mL) extraction was done and both phases collected and supplemented with methanol (200 µL). Anhydrous Na_2_SO_4_ was added to eliminate residual water. Chloroform was evaporated under nitrogen and the residue solubilized with 100 µL of toluene, 1960 µL of methanol, and 40 µL of concentrated H_2_SO_4_. The reaction mixture was incubated at 90 °C for 2 h to form fatty acid methyl esters. At the end of the reaction, 1 mL of 5% NaCl and 2 mL of hexane were added, stirred, and the organic phase recovered. Hexane (2 mL) was added to the aqueous phase and both organic phases pooled and evaporated under nitrogen. This residue was sealed and stored at –20 °C. All steps were performed at 4 °C and all organic solvents were mixed with 0.002% (*w:v*) butylated hydroxytoluene (2, (6)-di-tert-butyl-*p*-cresol) (BHT). To dissolve the fatty acid methyl esters residue from the extract, 100 µl of hexane were added to the dry residue. The analysis was performed with a gas chromatograph Carlo Erba (Milan, Italy) coupled with FID (flame ion detection) and with a column of CP SIL 88 (Agilent, Santa Clara, CA, USA) using an injector temperature of 230 °C, an oven temperature of 190 °C, and a helium gas vector of 1 mL/mm. The chromatogram was analyzed with the Chrompac-Running computer software from Varian (Palo Alto, CA, USA).

### 4.9. Detection of Complex Sphingolipids by TLC

Membrane suspensions were treated as in Castegnaro et al. [69] for lipid extraction supplementing *D*-sphingosine or Ceramide 6:0, as internal standards. The resulting lipid residue was resuspended and applied to 60F 254 TLC plates (Merck, Darmstadt, Germany) and eluted with a mobile phase of CHCl_3_:CH_3_OH:CH_3_COOH, 94:1:5, *v:v:v* [70] for ceramide detection and with CHCl_3_: CH_3_OH:CaCl_2_ 0.22 %, 60:35:8, *v:v:v* for glucosylceramide detection [71]. Plates were then developed with a cupper solution [72].

### 4.10. Immunoblotting Assays

Proteins from membrane fractions were resolved in 1 mm thick tricine-SDS PAGE using 10% T, 6% C and 10%, 3% gels for PM H^+^-ATPase and 14-3-3 protein separation, respectively, and electrophoresed at 60 v for 0.5 h and 90 v for 2 h in a Mini-Protean II System BioRad (Hercules, CA, USA). Then, gels were electro-transferred to a polyvinylidene difluoride (PVDF) membrane Immobilon, Millipore (Billerica, MA, USA) in a chamber TE22 Mightly Small Transfer Tank, Hoefer (Holliston, MA, USA) containing 15 mM phosphate buffer, pH 6.9, 20% methanol and 0.05% SDS at 25 v for 2.25 h. The PVDF membrane was incubated with the first antibody (antibody vs. PM H^+^-ATPase was used in a 1:4000 dilution and antibody vs. 14-3-3 protein was used in a 1:1000 dilution) in a TBS buffer containing 500 mM NaCl, 20 mM Tris-HCl (pH 7.5) and 2% skimmed milk (overnight at 4 °C for the anti H^+^-ATPase antibody or at room temperature for the 14-3-3 antibody). The second antibody (goat antirabbit IgG alkaline phosphatase conjugate) was used in a 1:1000 dilution in TBS buffer containing 5% skimmed milk, incubating the PVDF membrane for 2 h at room temperature. The reaction was developed as previously reported [73].

### 4.11. Protein Determination

This was done according to the procedure of Peterson [74] using BSA as standard.

### 4.12. Statistical Analyses

Experiments were carried out at least from three biological samples. Where indicated, experimental data were statistically analyzed by the Origin program, version 4.10 (Microcal sofware) and with LSD (T) comparison of means using the Statistix Version 4 Analytical software (Tallahassee, FL, USA). 

## Supplementary Materials

The following are available online at https://www.mdpi.com/2223-7747/9/2/150/s1. Figure S1: Linearity of the ATP hydrolysis assay as a function of time. Table S1: Levels of total protein in subcellular fractions from maize embryos exposed to FB1.
